# A Bilayer High-Temperature Dielectric Film with Superior Breakdown Strength and Energy Storage Density

**DOI:** 10.1007/s40820-023-01121-6

**Published:** 2023-06-08

**Authors:** Jiang-Bo Ping, Qi-Kun Feng, Yong-Xin Zhang, Xin-Jie Wang, Lei Huang, Shao-Long Zhong, Zhi-Min Dang

**Affiliations:** State Key Laboratory of Power System, Department of Electrical Engineering, Tsinghua University, Beijing, 100084 People’s Republic of China

**Keywords:** Film capacitor, Dielectric property, Boron nitride nanosheets, Surface coating, Energy storage characteristics

## Abstract

The further electrification of various fields in production and daily life makes it a topic worthy of exploration to improve the performance of capacitors for a long time, including thin-film capacitors. The discharge energy density of thin-film capacitors that serves as one of the important types directly depends on electric field strength and the dielectric constant of the insulation material. However, it has long been a great challenge to improve the breakdown strength and dielectric constant simultaneously. Considering that boron nitride nanosheets (BNNS) possess superior insulation and thermal conductivity owing to wide band gap and 2-dimensional structure, a bilayer polymer film is prepared via coating BNNS by solution casting on surface of polyethylene terephthalate (PET) films. By revealing the bandgap and insulating behavior with UV absorption spectrum, leakage current, and finite element calculation, it is manifested that nanocoating contributes to enhance the bandgap of polymer films, thereby suppressing the charge injection by redirecting their transport from electrodes. Worthy to note that an ultrahigh breakdown field strength (~ 736 MV m^−1^), an excellent discharge energy density (~ 8.77 J cm^−3^) and a prominent charge–discharge efficiency (~ 96.51%) are achieved concurrently, which is ascribed to the contribution of BNNS ultrathin layer. In addition, the modified PET films also have superior comprehensive performance at high temperatures (~ 120 °C). The materials and methods here selected are easily accessible and facile, which are suitable for large-scale roll-to-roll process production, and are of certain significance to explore the methods about film modification suitable for commercial promotion.

**Supplementary Information:**

The online version contains supplementary material available at 10.1007/s40820-023-01121-6.

## Highlights

- A bilayer dielectric film is prepared via coating boron nitride nanosheets (BNNSs) by solution casting on the surface of polyethylene terephthalate (PET) film.
- The BNNS layer acts as the efficient barrier layer to suppress the charge injection, thereby making the surface-modified PET films exhibit excellent breakdown strength and electrostatic energy storage performance.
- The surface coating methods are accessible and suitable for large-scale roll-to-roll process production of dielectric films.

**Supplementary Information:**

The online version contains supplementary material available at 10.1007/s40820-023-01121-6.

## Graphical abstract

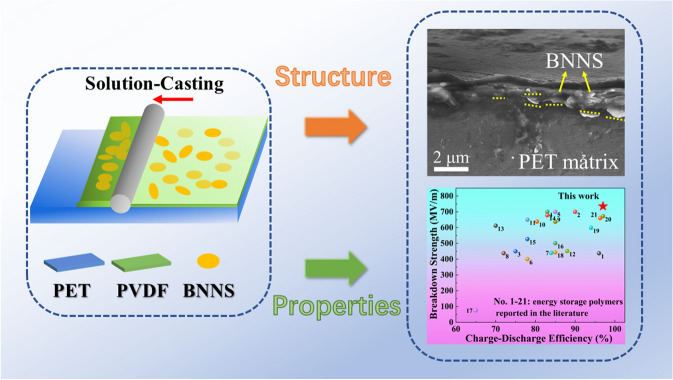

## Introduction

At present, the fossil energy crisis and global warming are still severe problems that mankind has to face together and new energy needs to be developed and utilized urgently. Therefore, the development of new energy will usher in unprecedented opportunities, including new energy power generation and grid connection, the promotion and popularization of new energy vehicles, and many other fields that need film capacitors [1–5]. As one of the three main types of capacitors, thin-film capacitors have been widely used because of their low loss, high withstand field strength and excellent operation reliability. However, its low energy density makes volume often large, which is not conducive to its application in some scenarios with strict space restrictions such as electric vehicles and electromagnetic pulse devices. Therefore, improving the energy storage density of thin-film capacitors has an important application value. For linear dielectric materials (such as polyester film), the dielectric constant remains almost unchanged with the change of the applied electric field [6, 7]. The expression of energy storage density is shown as follows: *W* = 1/2DE = 1/2 ${\varepsilon }_{0}{\varepsilon }_{r}{E}^{2}$, where *W* is the energy density, *E* is the electric field strength, and *D* is electric displacement, ${\varepsilon }_{0}$ and ${\varepsilon }_{r}$ represent the vacuum dielectric constant and the relative dielectric constant of the material, respectively. And, ${\varepsilon }_{0}\hspace{0.17em}$= 8.85 × 10^–12^ F m^−1^ is a constant. Obviously, improving the dielectric energy storage density can be set from two aspects: improving the dielectric constant and enhancing the working field strength, and the latter is a more effective strategy because of a square effect compared with the former [8–11].

In the process of preparing organic dielectric thin films, adding modified inorganic fillers to improve the overall performance of materials is the most direct method. The fillers used include zero dimensional granules, one-dimensional fibers (or tubes), and two-dimensional (2-D) flakes [12–16]. For instance, Li et al. incorporated ultra-thin boron nitride nanosheets in the ferroelectric terpolymers via solution method and the breakdown field of the polymer nanocomposites get a great enhancement. They found the reason for this great achievement is that BNNSs serve both as a tough frame and an insulating barrier. However, compatibility problems exist between the organic phase and inorganic phase for the polymer film incorporating inorganic fillers [16]. Nevertheless, considering that the bad compatibility between different phase materials in multiphase blend composite films tends to harm the flexibility of polymer film, many researchers choose the design scheme of multilayer structure films [17, 18]. The micro-laminate composites using reduced graphene oxide (rGO) and BNNS embedded in different matrices (PI: polyimide, PU: polyurethane) have been investigated by Kim and co-workers [19, 20]. Excellent dielectric and thermal properties could be achieved by the rational assembly of functional nanofillers. Huang et al. cleverly used the shear force in the scraping process to rehearse BNNS into a compact and continuous interlayer to prepare a multilayer composite with polyvinylidene fluoride (PVDF) outer layer and BNNS middle layer. The interlayer greatly reduces the local electric field distortion and prevents the propagation of electric tree, endowing the composite with high breakdown performance [21]. However, the interlayer and the polymer outer layer face the risk of relative displacement when the film undergoes the winding process.

Attractively, some recent studies paid more attention to the contact surface between the electrode and dielectric materials [22–26], which have been proven to be decisive for the insulating properties of the dielectric films. For example, Pei et al. inserted polymethylmethacrylate (PMMA) nanolayer at the interface between an electrode and original dielectric to improve surface defects and Young's modulus. On the premise of not sacrificing efficiency, all organic double-layer dielectric materials with high breakdown strength (767.05 MV m^−1^) were prepared (original PVDF was 637.31 MV m^−1^) [22, 24]. The high cost and unacceptable dielectric loss severely limit the wide application of PVDF matrix. In addition, Zhou and co-workers fabricated dielectric biaxially oriented polypropylene (BOPP) films coated with SiO_2_ via plasma-enhanced chemical vapor deposition, which improved the potential barrier at the electrode/dielectric interface and suppressed the charge injection [27]. Nevertheless, the performance of modified polymers at high temperatures still needs to be further improved.

The research idea of improving the breakdown performance of materials starts from two aspects: the bulk properties of the material and the interface between the material and the electrode, and the latter has gradually attracted the attention of researchers. In the present work, this situation is caused by further understanding the breakdown characteristics of materials and consideration about whether the modification method has the potential for large-scale production [28, 29]. In this work, the most common and convenient method is adopted as fellows. A layer of film including BNNSs is coated on a commercial PET film by solution casting method, and BNNSs are aligned parallel to the film by making full use of fluid shear force to prepare composite films with improved breakdown performance. The gas barrier abilities, thermal conductivity and mechanical properties of PET composites incorporated with BNNS have been studied [30–32]; however, little research has been conducted on insulation and energy storage performance. Due to presenting a two-dimensional graphene-like structure, the BNNS nanosheets possess excellent insulation (bandgap width of about 6.0 eV, breakdown field strength of about 800 MV m^−1^) and high thermal conductivity of 390 W (m K)^−1^. Therefore, it is widely used in the field of improving the insulation and thermal conductivity of materials [33–35]. Considering the powder state of BNNSs at room temperature, the dielectric constant of composite films, and the combination between coating layer and PET matrix, the PVDF was selected as the dispersion of BNNSs and binder of the coating layer and PET matrix. The results show that the BNNSs layer improves the barrier height and blocks the carrier injection, so as to improve the breakdown performance of PET. As a result, the bilayer polymer film also displays excellent energy storage characteristics.

## Experimental Section

### Materials

The BNNSs with an average lateral size of 0.1–0.4 μm were purchased from XFNANO Technology Co., LTD (China). N, N-Dimethylformamide (DMF) (AR grade) was provided from Tansoole (China). PVDF powder was purchased from Shanghai 3F New Materials Technology Co., LTD (China). PET film with a thickness of 11.8 μm was purchased from Anhui Tongai Electronic Material Co., Ltd (China). BOPP film was purchased from Guangdong Jiangmen Runtian Co., Ltd (China). PEI film was purchased from PolyK Technologies (USA). Pyromellitic dianhydride (PMDA) and 4,4′ diamino diphenyl ether (ODA) for the preparation of PI film was supplied by Innochem Co., LTD.

### Preparation of the Modified PET Films

Firstly, the solution of PVDF and DMF was prepared by adding 0.6 g of PVDF powder after being dried at 60 °C for 24 h to 12 g of DMF and stirring with magnetic force at 600 rpm at 50 °C for 12 h. Then, blended certain amounts of BNNS powder after being dried at 60 °C for 24 h and 20 g of DMF, and sonicated the mixture by a 3 h tip-type sonication (100 W, 300 W × 33.3%). Next, stirred the solution of PVDF-DMF and mixture of BNNS-DMF by magnetic force at 600 rpm at 50 °C for 8 h to acquire the uniform dispersion. Finally, cast the mixture dispersion on one piece of PET film on glass plates by a doctor blade and dried the modified films at 70 °C for 12 h. In order to study the effect of BNNS content on the comprehensive properties of modified PET films, the thickness of coating layer was kept constant while the content of BNNSs in PVDF was changed. In addition, under the premise of keeping the BNNS content constant (2.67 vol%), the influence mechanism of coating thickness on the properties of modified films was studied by further changing the thickness of coating layer.

### Microstructural Characterization

Surface morphology and cross-section morphology of modified films and BNNSs sputtered by conducting carbon were observed with a Zeiss Merlin emission electron microscope. The transmission electron microscopy (TEM) images and corresponding diffraction patterns of BNNSs were observed with JEM-2010 (Japan). Atomic force microscopy (AFM, BRUKER Dimension Icon) was used to investigate the size of BNNSs. An optical microscope (LEIKA, MC190 HD) was utilized to study the optical appearances near the breakdown point of films. Dynamic mechanical analysis (DMA) was measured by a DMA 850 system (TA Instruments, United States) with a heating rate of 3 °C min^−1^.

### Measurements of Electrical and Capacitive Properties

The modified films for the electrical measurements were subjected to sputtering treatment on both sides for a gold electrode of a diameter of 2 mm and a thickness of about 50 nm with ETD-900 M Magnetron Sputtering Apparatus. Dielectric constant and loss were measured using a 4294A Precision Impedance Analyzer from Agilent Company (USA) and a SU-261 temperature control box from ESPEC Company (Japan). Conductivity was measured under distracting electric field provided by 2290-10 10 kV POWER SUPPLY and 2635B SYSTEM Source Meter both from KEITHLEY Company (USA). For the test, the initial field strength is 20 kV mm^−1^, the step size is 20 kV mm^−1^, the termination is 300 kV mm^−1^, and each field strength value is maintained for 900 s to get the current steady. D-E loops were acquired by a Precision Multiferroic test system (Radiant Technologies) and the samples were subjected to triangular unipolar waves with a frequency of 10^2^ Hz. Breakdown strength measurements were performed on BOHER High Voltage Power Supplies 72030P under a DC voltage ramp of about 500 V s^−1^ and a maximum current of 1.5 mA. The charge–discharge cycle test, as well as the power density measurement, was performed using PK-CPR1502 (USA). The samples were immersed in silicone oil for tests about D-E loops, breakdown strength and charge–discharge cycle. The high voltage power source (Agitek, DG4012) was used to measure the self-healing properties of metalized film, and the voltage as well as leakage current waveforms are recorded by an oscilloscope (LECROY, HPO5041). The UV absorption spectrum was performed on HITACHI U-3900 (Japan).

## Results and Discussion

### Fabrication and Morphology of Layer-structured Films

**Fig. 1:**
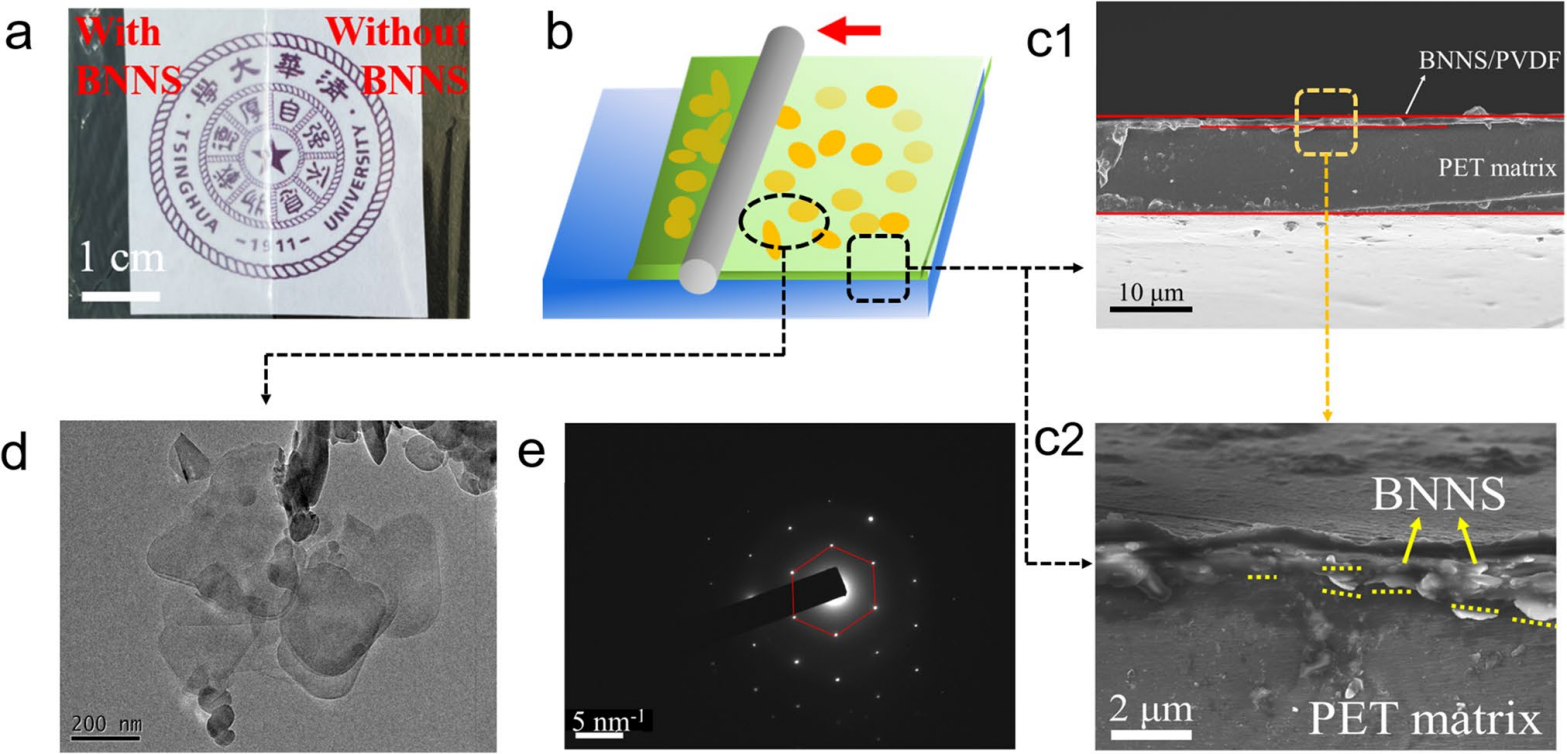
**a** Macroscopic appearance of the modified PET film and pure PET film. **b** Diagram about the fabrication of modified film. **c** Cross-sectional morphologies of the modified PET film in SEM: (1) holistic drawing of modified PET film, (2) partial enlarged drawing of BNNS layer. **d** TEM image of BNNSs. **e** SAED pattern of BNNSs

By rationally. The modified PET films were fabricated by solution casting schematically illustrated briefly in Fig. 1b (Fig. 1a is the macroscopic appearance of the modified and pure PET films) and described in detail in the Experimental Section. Firstly, the micro-morphology and structure of the BNNSs powder were analyzed by SEM and TEM, respectively. As shown in Figs. 1d and S1, the utilized BNNSs in this research present as round or oval flakes with sheet diameters of 100–400 nm which we assumed the BNNSs could provide preconditions as a shielding layer for carrier transmission. In addition, Fig. S1b illustrates the TEM image of vertical BNNS with a thickness of 10–20 nm roughly. Figure S1c shows that the thickness of BNNS is around 40 nm, which is caused by the overlapping of multiple BNNS layers. Concerning the selected area electron diffraction (SAED) pattern displayed in Fig. 1e, we deduced that the BNNSs are hexagonal structure. Then, in order to know the macroscopic appearance, the optical images of pure PET film and the modified PET film are shown in Fig. 1a. In stark contrast to the transparent pure PET film macroscopically, the modified PET film looks evenly white as a result of the uniform distribution of BNNSs.

In particular, the cross section of the modified PET film was observed by SEM, to observe more subtle structural characters after coating treatment. The coating layer possesses a uniform thickness of about 1 μm and good bonding with the matrix PET film (Fig. 1c). When zooming in the view of the cross section, it was difficult to see the BNNS incorporated in coating layer. Good contact is one reason BNNSs could not be easy to be observed and the other one is the excessively small thickness of BNNSs which the aspect of the BNNS flake is inclined to assemble parallel to the film surface due to shear force during the coating process. Fortunately, several pieces of BNNSs leaking on the surface could be found as shown in Fig. 1c2. Furthermore, in terms of observations, the configuration of BNNSs is almost parallel to the plane of film indeed which plays an important role on shielding the charge carrier we will state thereinafter.

### Electroinsulating Properties of Layer-Structured Films

**Fig. 2:**
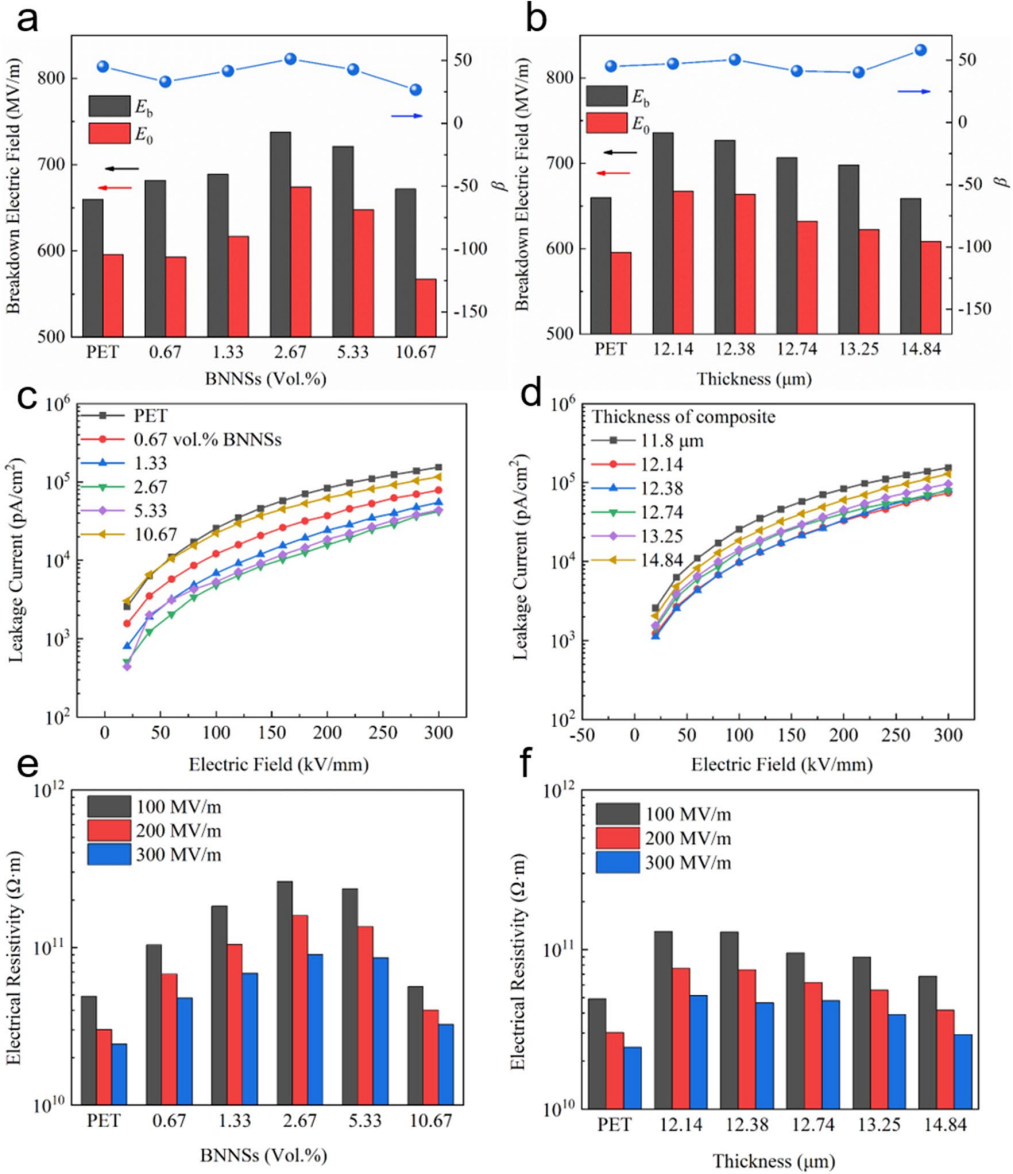
The insulating performances of the two groups modified PET films with different contents, and different thicknesses. **a**, **b** Breakdown field and shape parameter *β*. **c**, **d** Leakage current under different applied electric fields. **e**, **f** Volume resistivity under different applied electric fields

As we mentioned, the energy density of thin film capacitors is determined by the dielectric constant and breakdown strength simultaneously, with the impact of breakdown strength being greater. In addition, the breakdown strength of dielectric polymers also plays a decisive role in the safe operation of film capacitors. The breakdown performance of the modified PET films is estimated with a two-parameter Weibull distribution function as follows:

1 $$P(E) = 1 - \exp \left[ { - \left( {\frac{E}{{E_{{\text{b}}} }}} \right)^{\beta } } \right]$$

where *P*(*E*) is the cumulative probability of the electric breakdown failure, *E* the electric field strength, *E*_b_ the scale parameter which means breakdown occurs in the sample tested in the probability of 63.2% and can be as the characteristic breakdown strength of dielectrics [11]. The shape parameter *β* shows the dispersion of test data on *E* and the quality of dielectric films to a certain extent. Meanwhile, the field strength recorded as *E*_0_ when the breakdown occurs in 1% probability was also calculated in this work. *E*_0_ could be used to roughly characterize the upper limit of the safe use of the modified PET films. The Weibull distribution of the breakdown electric field of the modified PET films is given in Fig. S2a. Firstly, compared with the pure PET film, the *E*_b_ of the modified PET films coated with different content of BNNSs achieves enhancement to different extents (Fig. 2a, b). From Fig. 2a, the improvement effect of *E*_b_ of the modified PET films increases first and then decreases with the increase in BNNSs contents. The obvious improvement of *E*_b_ based on the pure PET film (from 659.9 to 737.6 MV m^−1^, increasing by 11.8%) emerges in the PET/BNNS-2.67 vol% sample. Moreover, the *E*_b_ of PET/BNNS-2.67 vol% bilayer film exceeds other modified PET films with different content BNNSs distinctly, which indicates 2.67 vol% most closely approaches the optimal value of BNNSs content. The fact that the *E*_b_ and content of BNNSs are not monotonous shows that the content and distribution of BNNSs jointly determine the breakdown properties of bilayer composite films. A small amount of BNNSs cannot form an effective barrier layer to suppress charge injection, while excessive addition of BNNSs may cause distortion of local field strength inside the polymer film and a decrease in mechanical properties [16]. The variation about *E*_0_ of the modified PET films with different content of BNNSs almost resembles *E*_b_ and this scene means the optimized composite films can endure a higher electric field than the pure PET film in application. Meanwhile, there is no significant difference in regularity of the modified PET films (characterized via *β* that the smaller *β* represents the worse dispersibility) after being coated (Figs. 2a and S2a), which declares a good uniformity of the coating layer and is coincident with Fig. 1c.

As is well known, the thickness of dielectric is a critical factor for insulation and breakdown. Therefore, there is often an order of magnitude difference in breakdown field strength between film and block [29]. Therefore, a group of modified PET films coated by BNNSs with different thickness was designed and analyzed, besides the group of films coated with different contents of BNNSs. The column diagram and Weibull distribution of the breakdown electric field of this group (different thickness) are given in Figs. 2b and S2b, separately. As seen in Fig. 2b, *E*_b_ has a significant negative correlation with the thickness (namely PET film of the same specification plus a coated layer of different thickness) of modified PET films. Compared with the pure PET film, the *E*_b_ of the thinnest modified film with the thickness of only 0.34 μm coating layer is the highest (736.06 MV m^−1^) versus that of the pure PET (659.90 MV m^−1^). The variation trend of *E*_0_ with thickness resembles that of *E*_b_, which shows the thinner coating layer makes a better impact on improving the working voltage and breakdown field strength of the composite films when the content of BNNSs is certain. It is clearly seen in Fig. S2b, the changes of *β* about the latter group of samples (different thickness) resemble the former (different contents of BNNSs).

After analyzing breakdown electric field of the modified films, the corresponding leakage current and volume resistivity under 100, 200, and 300 MV m^−1^ are stated, respectively, which both reflect the bulk insulation properties of bilayer film materials [36, 37]. Taking PET/BNNS-0.67 vol% for example, the current density of all samples increases exponentially but steadily with the increase in electric field and this illustrates the samples keep insulated within the electric field we analyzing (Fig. S3). Gratifyingly, excellent consistency with the breakdown performance can be seen in the trend of the leakage current and volume resistivity in Fig. 2c–f. The leakage currents show the resistive current formed by the movement of carriers within dielectrics under the action of an external electric field, which could be used to reflect the insulating properties of dielectric materials. Both the sample PET/BNNS-2.67 vol% in Fig. 2c and the sample PET/BNNS-d12.14 in Fig. 2d retain the lowest leakage current from start to finish in their respective tests. In terms of volume resistivity, it raises evidently from 0.32 × 10^11^ Ω m of PET film to 1.60 × 10^11^ Ω m of PET/BNNS-2.67 vol% under 200 MV m^−1^, likewise, to 0.76 × 10^11^ Ω m of PET/BNNS-d12.14. This strongly supports the previous explanation of how the content of BNNSs or the thickness of the coating layer affects breakdown performance. The optical appearance near the breakdown point of the sample reflects the above conclusion to a certain extent (Fig. S4). These through-holes were caused during electric breakdown tests and indicated that the film has self-healing characteristics before and after being coated. In stark contrast with the pure PET film where the evaporated electrode area is nearly the same as the lost dielectric area (Fig. S4a), the modified film lost a larger electrode area than dielectric (Fig. S4b). The phenomenon presented by the latter reflects two facts: one is that a higher electric field when breakdown took place, and the other is that the coating layer changed the heat dissipation direction, which demonstrated an effective function layer in the coating layer played the role of carriers shielding and heat dissipation [21]. Under the pulse voltage, the changing process of current and voltage in the self-healing tests of the modified PET film is shown in Fig. S4c, which proves that the modified films have excellent self-healing ability and operating stability.

To analyze stationary electric field distribution through the coating layer, omitting the matrix with a highly uniform structure to display the results more significantly, finite element analysis (FEA) was conducted. The stereoscopic distribution of BNNS in the coating layer is displayed vividly in Fig. S5a–f. The size of the simulation model in Fig. S6a–c is 1 μm × 1 μm × 1 μm, and that in Fig. S6d–f is same bottom area and different thickness. The external field strength borne by the above six models is 500 kV mm^−1^. As shown in Figs. S6a, b, comparing the perspective drawings with an aerial view, insufficient BNNSs are not enough to form an effective carrier shielding layer as well as a thermal diffusion layer. When the appropriate amount of BNNSs is added, although there is also an internal distortion of electric field, the BNNSs barrier layer plays a dominant role in improving the insulation performance, and the breakdown strength of the modified PET film could be improved. However, from the perspective of the cross section of the coating layer (in Fig. S6c), the agglomeration of BNNSs at high contents may lead to more serious distortion of electric field, which reduces the breakdown strength of the modified films. In addition, for the thinner coating layer, BNNSs are subjected to the stronger shear force which makes smaller angles between the flakes and the plane of film. And, BNNS presents distinct different electric and thermal performances, resulting from its 2-D structure. So, a smaller angle means a larger effective shielding area for carriers and more prominent heat dissipation along the plane of films. Besides, when the BNNSs content remains constant (2.67 vol%), the thickening of the coating layer may result in some BNNSs no longer being distributed along the in-plane direction, which is not conducive to inhibiting charge injection. Thus, appropriate contents of BNNSs and thin coating layer both make contributions to the effective function layer from different directions, one with the plane of the film, the other with the cross section of the film, respectively [35].

### Dielectric and Energy Storage Capabilities of Layer-Structured Films

**Fig. 3:**
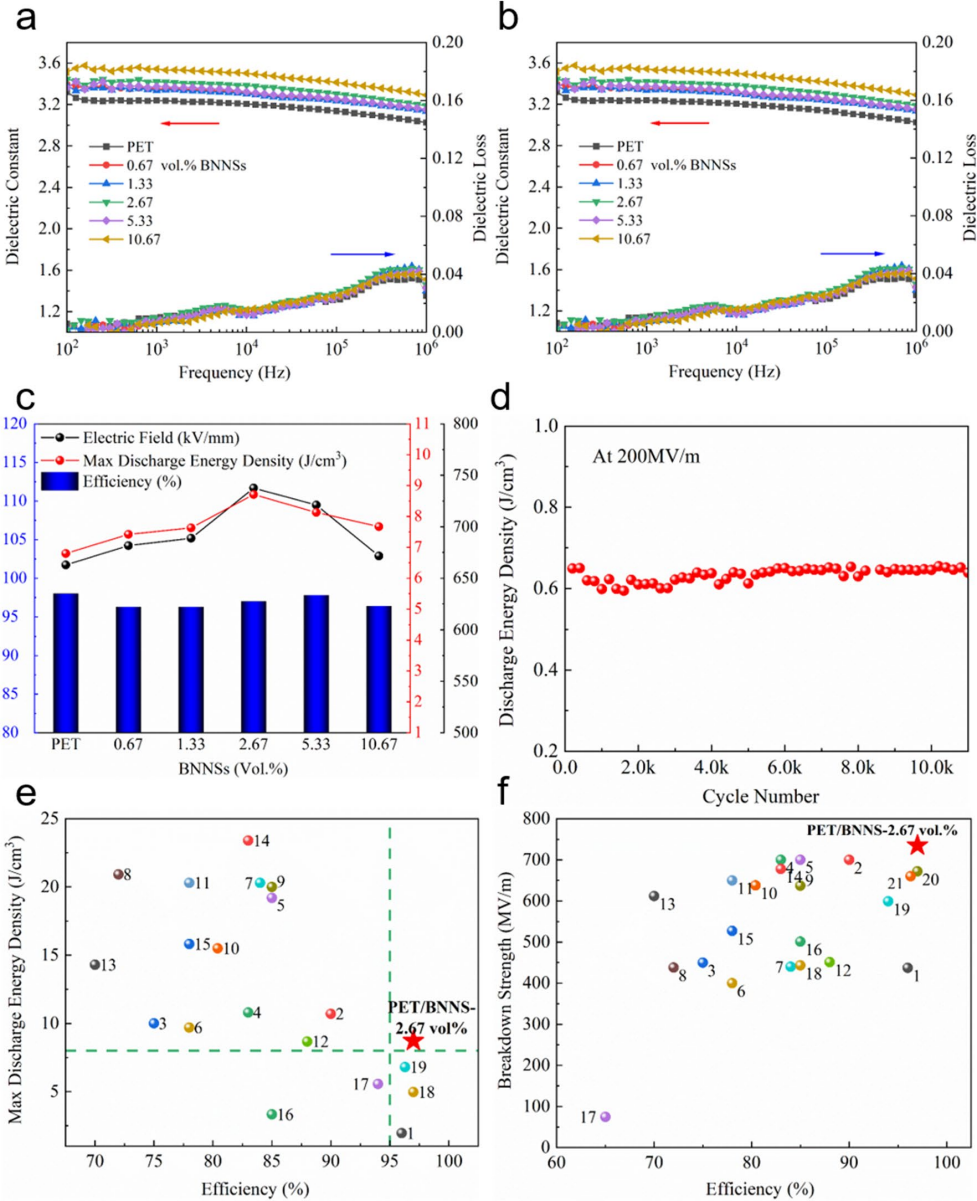
**a** Frequency dependence of dielectric constant and loss tangent at 20 °C. **b** Temperature dependence of dielectric constant and loss at 10^3^ Hz. **c** Discharge energy density and efficiency of the modified films with different content of BNNSs. **d** Cycle performance of PET/BNNS-2.67 vol%. **e**, **f** Comparison among dielectric polymer nanocomposites with existing literatures (The label 1 to 19 represents the materials listed in Table S1)

The enhancement and causes of the breakdown performance of composite films have been elaborated above. Then, dielectric constant and loss possessing major importance for dielectric materials, and discharge energy density and efficiency which are critical parameters for film capacitors, will be discussed. The dielectric constant (*ε*) and loss (tan* δ*) of composite film samples were tested in the frequency range of 10^2^–10^6^ Hz at 20, 40, 60, 80, 100, and 120 °C, respectively. About the two groups of modified films, the frequency-dependent dielectric constant and dielectric loss at 20 °C are illustrated in Fig. 3a (different BNNSs contents) and Fig. S7a (different thicknesses), the temperature-dependent dielectric constant and dielectric loss at 10^3^ Hz in Fig. 3b (different BNNSs contents) and Fig. S7b (different thicknesses), respectively. As shown in Figs. 3a and S7a, all of the two groups of samples own stable dielectric constant with slight fluctuation less than 0.2 from 10^2^ to 10^6^ Hz. Compared with the pure PET film, the dielectric constant of other samples in the two groups is enhanced in varying degrees, with a maximum enhancement of 0.45 for PET/BNNS-d14.84 in the second group at 10^3^ Hz. The increase in dielectric constant of all samples is contributed to the coating layer with essential component PVDF, meanwhile restricted enhancement of dielectric constant is limited by the thickness of coating layer. Regardless of what BNNSs contributed to the increase in dielectric constant, BNNSs own close dielectric constant with PVDF and account for a small proportion in modified film. In addition, there’s also no significant numerical difference in the frequency-dependent of dielectric loss of the group of composite films coated BNNSs with different contents. This phenomenon could be explained by the fact that the PET matrix possesses excellent interfacial compatibility with the BNNS coating layer. Besides, the improvement in insulating properties caused by BNNS coating also contributes to suppressing dielectric losses. Similarly, the dielectric loss of the modified composite films with different thicknesses of coating layer shows little difference, except for PET/BNNS-d14.84 whose dielectric loss exceeds other samples evidently. The frequency-dependent dielectric loss of all samples displays the same trend that tan δ increased slowly first from 10^2^ to 10^4^ Hz and then significantly from 10^4^ to 10^6^ Hz. As shown in Figs. 3b and S7b, all samples retain good stability in *ε* and tan *δ* with the increase in temperature under 100 °C and start to increase gently around 120 °C. This phenomenon is caused by the matrix PET that there is a peak of tan *δ* near 120 °C when the molecular chain motion has a larger degree of freedom, which could be verified by the results of dynamic mechanical analysis (DMA, see Fig. S8).

To investigate the critical performance parameter of dielectrics for capacitors, the discharge energy density (*W*_d_) and efficiency (*η*) of the two groups of composite films are discussed. As shown in Fig. S9, the *W*_d_ of dielectric films can be calculated using applied electric field and electric displacement. Moreover, the *η* could be determined by the ratio of discharged and charged energy density. When subjected to electric fields of 200, 300, 400, 500, and 600 MV m^−1^ (Fig. S10), separately, the modified films coated by different contents of BNNSs present a small gap in *W*_d_ and *η* with them of the pure PET film. What contributed to the small numerical difference in *W*_d_ and *η* is the slight difference in *ε* between different samples. Furthermore, no matter the field strength changes, all the modified films display prominent *η* (almost all above 95%) due to the matrix PET film a linear dielectric material, which means excellent efficient energy conversion. As anticipated, the maximum discharge energy density (*W*_d-max_) achieves great improvement, which symbolizes the latent capacity of dielectric materials, like the breakdown performance of composite films. Compared with the pure PET film with *W*_d-max_ of 6.80 J/cm^3^, the *W*_d-max_ of PET/BNNS-2.67 vol% reaches 8.71 J cm^−3^ and exceeds 28.0% than that of pure PET film (see Fig. 3c). The largest *W*_d-max_ of 8.77 J cm^−3^ appears in PET/BNNS-d12.14 which achieves dramatically increment of 28.9% and still retains a prominent *η* of 96.51% (Fig. S7c). Furthermore, taking PET/BNNS-2.67 vol% for example, an excellent cycling performance is observed that it still holds stable discharge energy density with a fluctuation of less than 5% after 10,000 times of charge–discharge cycles under 200 MV m^−1^ (Fig. 3d). Power density results as a function of time before and after cycles indicate that the modified PET films own good cycle stability (Fig. S11). It is no exaggeration to say, PET/BNNS-2.67 vol% owns a huge superiority that holds relatively high discharge energy density and greater than 95% efficiency simultaneously. For this purpose, the comparison between some all-organic dielectrics, multilayer polymer dielectrics reported recently and pure polymer films currently which are possible to apply to large-scale production or have been commercially produced is exhibited in Fig. 3e–f. Evidently, the modified bilayer films prepared in this work have very high charge–discharge efficiency and excellent energy density. Particularly, it could be perceived from Fig. 3f that the modified PET films exhibit ultrahigh breakdown strength as well as efficiency. As for the film capacitor, disastrous failure usually results from the inferior insulating strength or the thermal runaway caused by low discharged efficiency. Fortunately, the modified bilayer films show outstanding performances of high *E*_b_ and efficiency, which makes modified PET films ideal candidates for dielectric film capacitors.

**Fig. 4:**
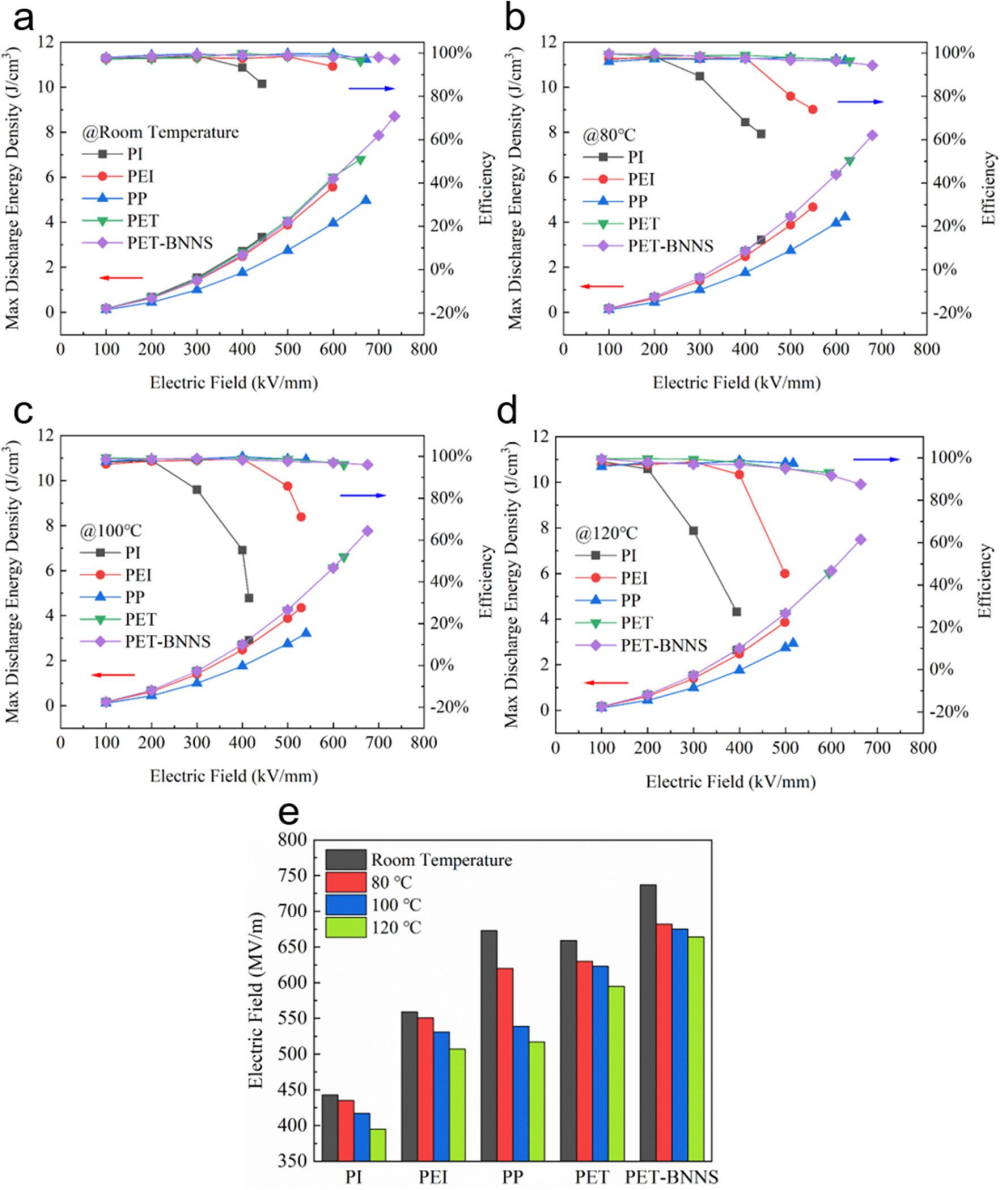
**a**–**d** Comparison of discharge energy density and efficiency, and **e** *E*_b_ of PI, PEI, PP, PET, and PET/BNNS-2.67 vol% (abbreviated as PET/BNNS) at room temperature, 80 °C, 100 °C, and 120 °C

Higher requirements are put forward for dielectric film capacitors in some harsh application scenarios with high ambient temperature, for gas drilling, electric vehicles and so on. So, research is in progress like a raging fire about polymer dielectrics with excellent electric and capacitive performance in a high-temperature environment of about 100–200 °C. In fact, biaxially oriented polypropylene (BOPP) film capacitors, which account for more than half of the market share, are recommended to be utilized below 85 °C and are restricted below 105 °C, owing to the poor thermal stability of BOPP. Polyimide (PI) and polyetherimide (PEI) are typically high-temperature resistant and have attracted more and more attention [38–42]. To research the performance of modified PET films at elevated temperature, the breakdown performance and energy storage performance of PET/BNNS-2.67 vol% at room temperature, 80, 100, and 120 °C are tested and the comparison with PI, PEI, PP, PET is illustrated in Table S2 and Fig. 4, concretely.

Due to PET possessing a higher recommended operating temperature than PP, PP shows obviously worse performance degradation in elevating temperature [27]. In terms of breakdown performance, *E*_b_ of PP has a maximum reduction of 23.19%, yet that of the other four are in a range of about 9% to 10%, from room temperature to 120 °C (Fig. 4e). The gap in *E*_b_ between PP and PET/BNNS-2.67 vol% directly contributes to the main gap in max energy density, while another part results from different permittivity of them (Figs. 4b–d). As for PI and PEI, both own lower breakdown fields (Fig. 4e) resulting from lower bandgap (Fig. S12b, PI for 2.87 eV, PEI for 3.39 eV and PET for 4.05 eV) at room temperature. However, we can see a dramatic drop in efficiency from Figs. 4b–d in pace with the increase in electric field and environmental temperature. Particularly, low efficiency means a lot of Joule heat would produce so far as to cause thermal runaway. Nevertheless, the PET/BNNS-2.67 vol% still holds an excellent performance at 120 °C with *W*_d-max_ of 7.50 J cm^−3^ and *η* of 87.5% and exhibits the best comprehensive performance whether at room temperature or high temperature (near 120 °C). Specifically, the *W*_d-max_ of PET/BNNS-2.67 vol% are 8.71, 7.86, 7.76, 7.49 J cm^−3^ at room temperature, 80, 100, and 120 °C, which are higher than PI, PEI, PP, and PET.

## Conclusions

In summary, by applying wide bandgap 2-D material BNNSs to act as an effective shielding layer on PET for blocking charge carrier, we designed and prepared a bilayer polymer film with superior breakdown strength (736 MV m^−1^) and energy storage density (8.77 J cm^−3^). The successful construction of interfacial regions between PET and BNNSs has been verified by different methods. It is demonstrated from obtained results that the surface coating with 2-D sheets is a resultful strategy for enhancing the bandgap of dielectrics and impeding charge transport, respectively. Meanwhile, the bilayer polymer film, namely modified FET polymer film, has superior comprehensive performance at high temperatures (near 120 °C) which is expected to be applied in more flexible scenarios. In addition, the facile and scalable approach is proved to be significantly effective and provides a reference for improving breakdown simply on a large scale.

### Supplementary Information

Below is the link to the electronic supplementary material.

Supplementary file1 (PDF 1383 KB)
