# Supplementary material for: A Bilayer High-Temperature Dielectric Film with Superior Breakdown Strength and Energy Storage Density
Source: Nanomicro Lett. 2023 Jun 8;15:154. doi: 10.1007/s40820-023-01121-6 (PMC10250289; doi:10.1007/s40820-023-01121-6)
Supplement: Supplementary file 1 — Supplementary file1 (PDF 1383 KB) [file 40820_2023_1121_MOESM1_ESM.pdf]

Supporting Information for

A Bilayer High-Temperature Dielectric Film with Superior Breakdown Strength and Energy Storage Density

Jiang-Bo Ping[1, †], Qi-Kun Feng[1, †], Yong-Xin Zhang[1], Xin-Jie Wang[1], Lei Huang[1], Shao-Long Zhong[1] and Zhi-Min Dang[1, *]

[1]State Key Laboratory of Power System, Department of Electrical Engineering, Tsinghua University, Beijing 100084, P. R. China

[†]Jiang-Bo Ping and Qi-Kun Feng Contributed equally to this work.

*Corresponding author. E-mail: dangzm@tsinghua.edu.cn (Zhi-Min Dang)

Supplementary Figures and Tables

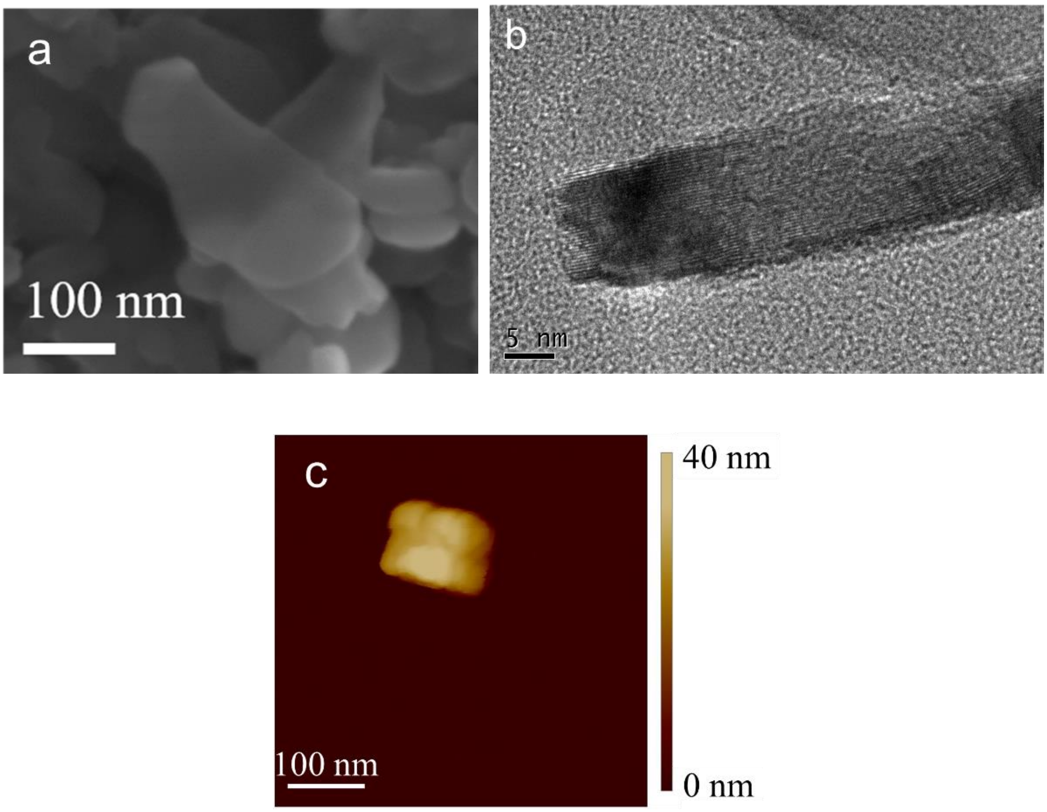

Fig. S1 (a) SEM morphology of BNNSs. **(b)** TEM image of vertical BNNS. **(c)** AFM image of BNNSs

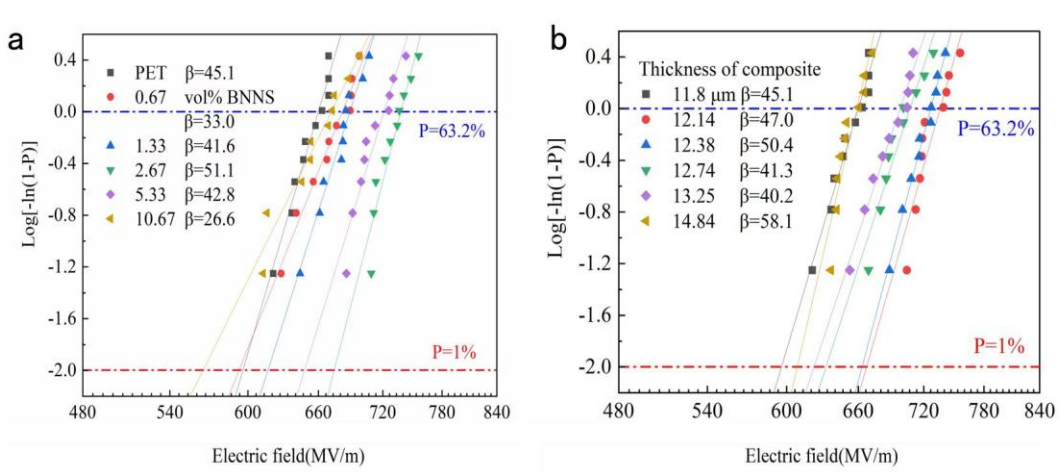

Fig. S2 Weibull distribution of the breakdown electric field of the modified PET films with **(a)** coated with 0.67, 1.33, 2.67, 5.33 and 10.67 vol.% BNNSs and **(b)** with different thicknesses in 11.8, 12.14, 12.38, 12.74, 13.25 and 14.84 μm

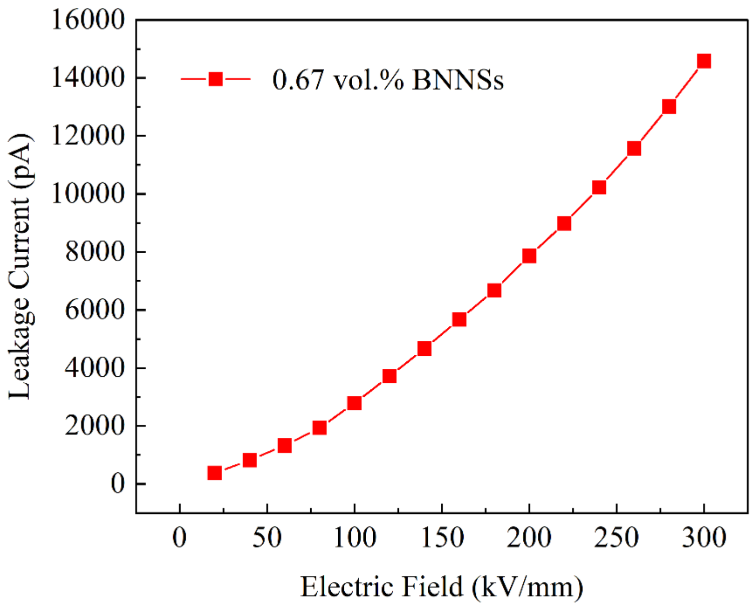

Fig. S3 The leakage current of the PET/BNNS-0.67 vol.% film under elevating electric field

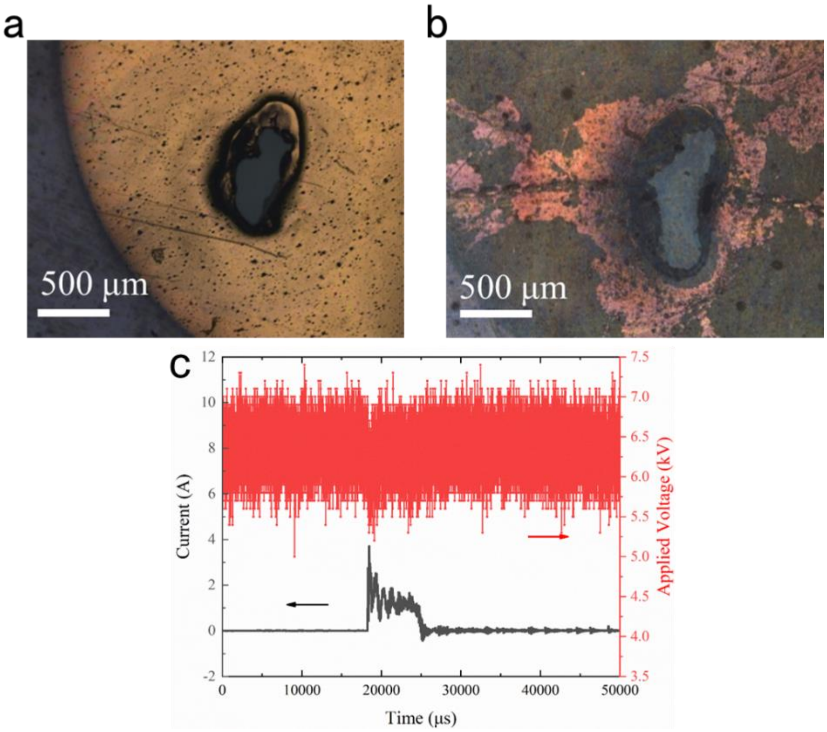

Fig. S4 The optical appearance near the breakdown point in **(a)** the pure PET film and **(b)** PET/BNNS-10.67 vol% film. **(c)** The current and voltage in the self-healing tests of modified PET film under a pulse voltage

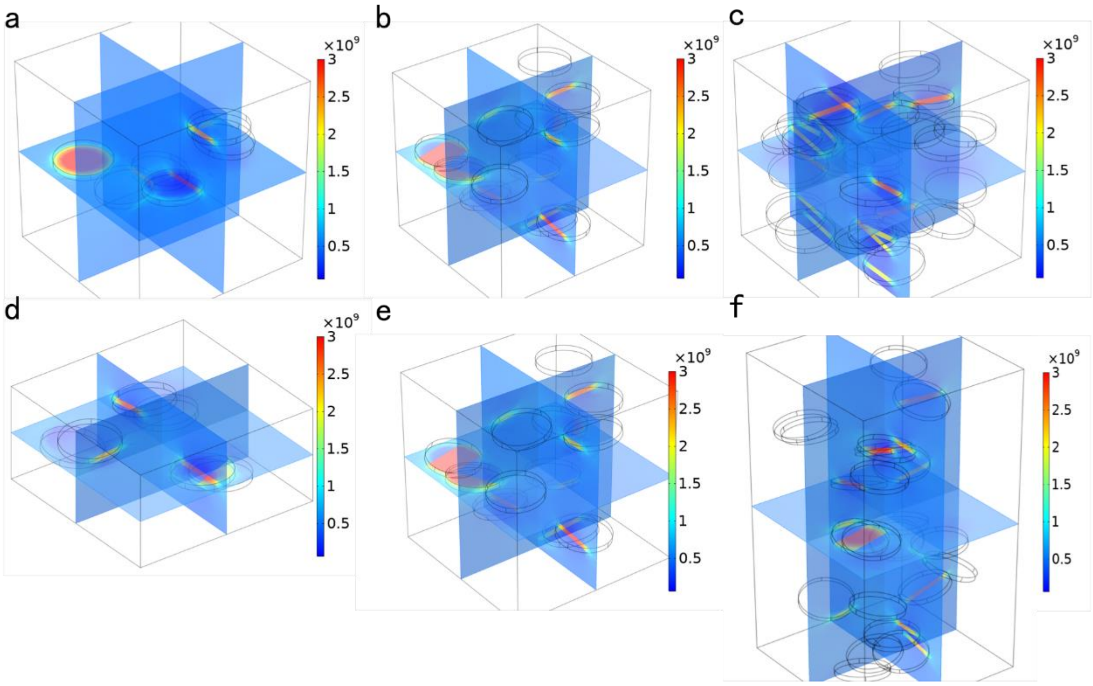

Fig. S5 The distribution simulated of BNNS in coating layer and corresponding E distribution of **(a)** PET/BNNS-1.33 vol.%, **(b)** 2.67 vol.% and **(c)** 5.33 vol.%, and in different thicknesses of **(d)** 0.5, **(e)** 1 and **(f)** 2 μm (the unit of all color legends is V/m)

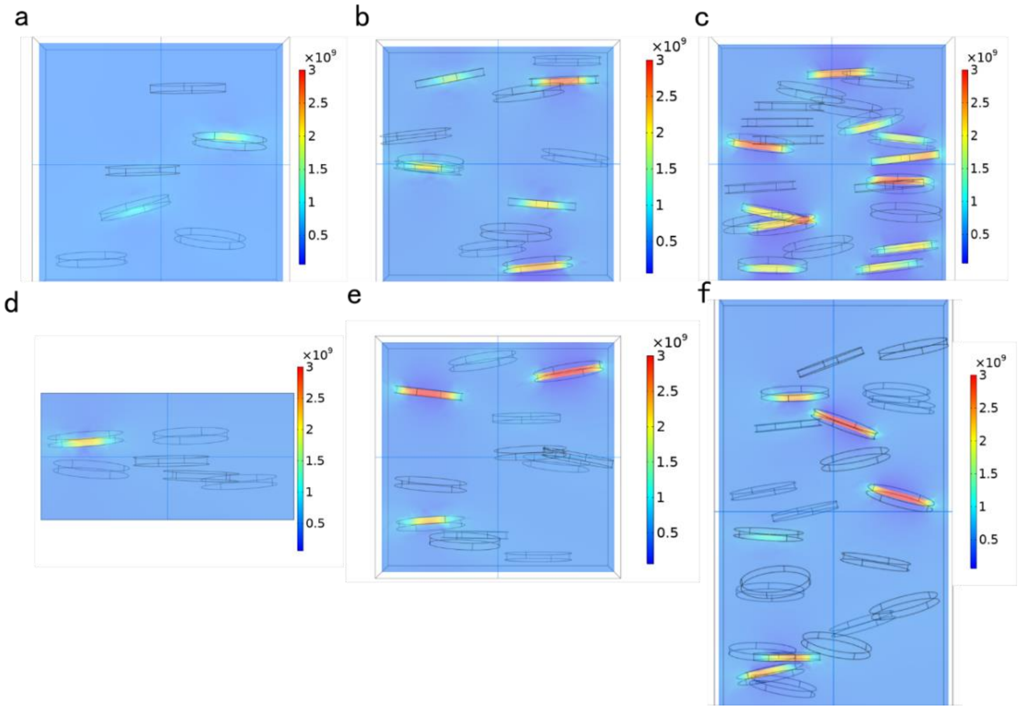

Fig. S6 Simulated transparency view and corresponding *E* distribution of **(a)** PET/BNNS-1.33 vol.%, **(b)** 2.67 vol.% and **(c)** 5.33 vol.%. And, in the same BNNS content of 2.67 vol.%, but with different **(d)** 0.5, **(e)** 1 and **(f)** 2 μm (The view of above 6 graphs is cross-section of coating layer)

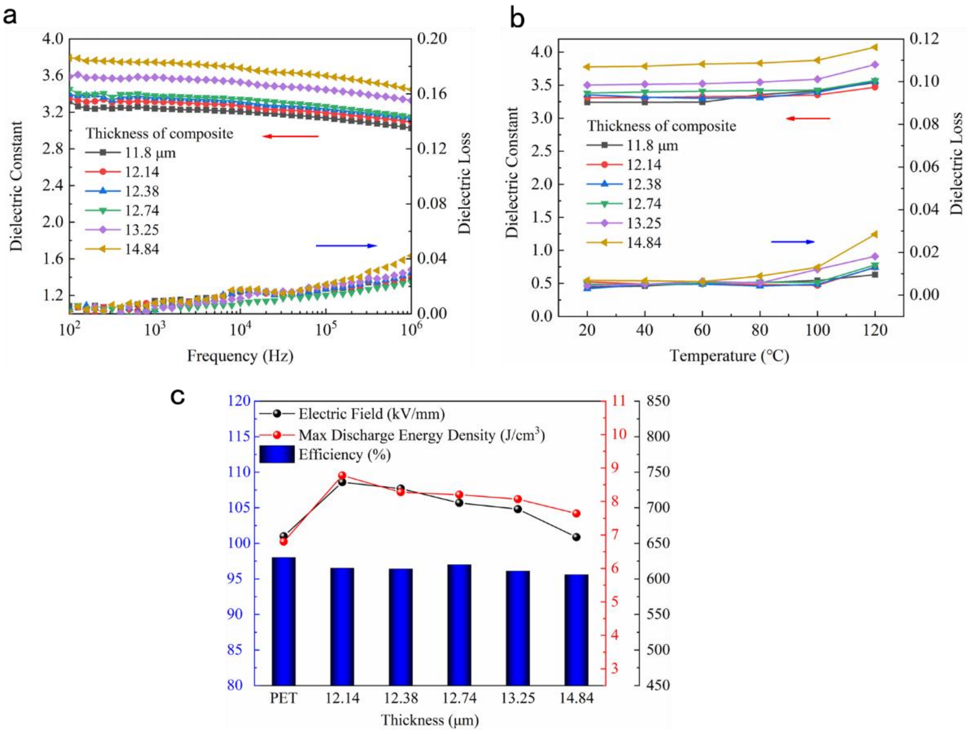

Fig. S7 **(a)** Frequency dependence of dielectric constant and loss at 20 °C, **(b)** temperature dependence of dielectric constant and loss at 10^3 Hz, **(c)** discharge energy density and efficiency of the modified films with different thicknesses of coating layer

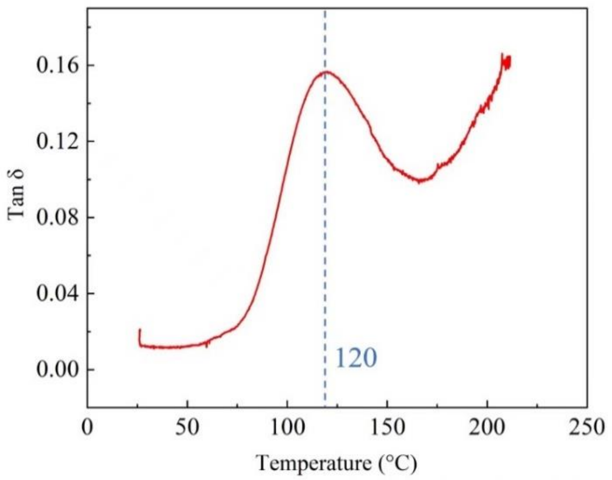

Fig. S8 The dynamic mechanical analysis result of PET film

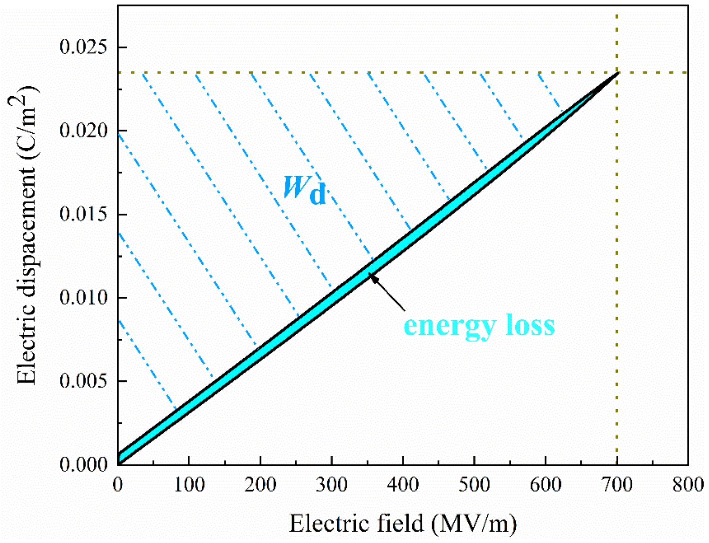

Fig. S9 The schematic diagram of D-E loop for dielectric film

The discharged energy density of dielectric films can be calculated using the following formula:

$$W_d = \int E dD \tag{S1}$$

The area enclosed by discharged curve and D-axis serves as the W_d, while the area enclosed by charged curve, discharged curve and D-axis is the energy loss (W_{loss}). Therefore, the working efficiency (η) during charge-discharge process can be determined by the following equation:

$$\eta = \frac{W_d}{W_d + W_{loss}} \tag{S2}$$

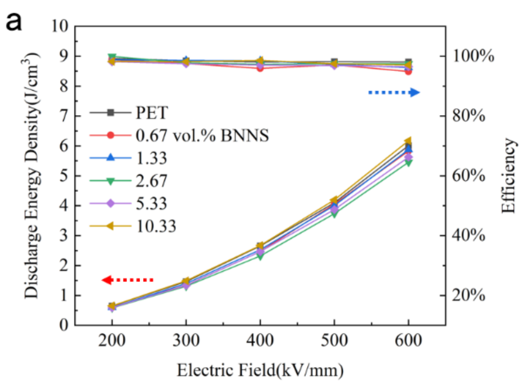

Fig. S10 Comparison of discharge energy density of the composite films **(a)** coated with different content of BNNSs and **(b)** different thicknesses

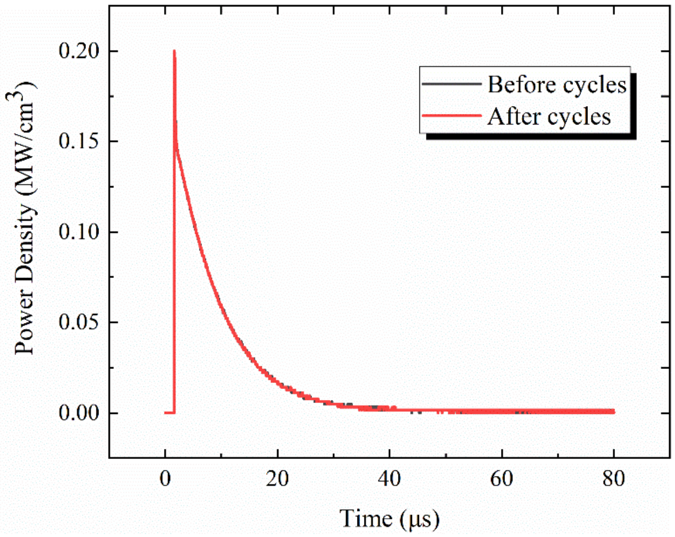

Fig. S11 Power density as a function of time of modified PET film before and after cycles

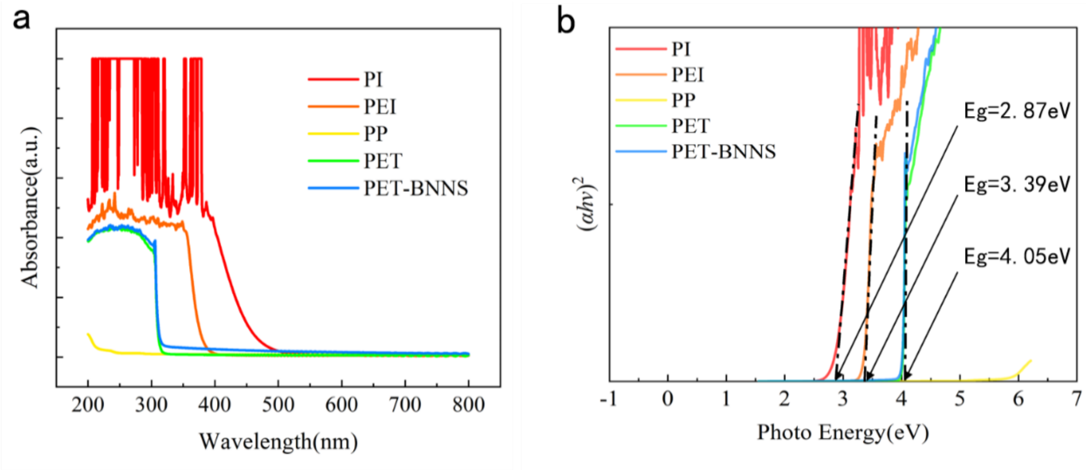

Fig. S12 (a) The UV absorption spectra and **(b)** $(\alpha h\nu)^2$ - hν plot of PI, PEI, PP, PET and PET-BNNS film

Table S1 Comparison of energy storage performances of polymer-based composites

Materials	Breakdown strength MV/m	Energy density J/cm^3	Energy efficiency %	code	References
MA-g-PP/PP (10/90 vol)	437.2	1.96	96	1	[S1]
PMMA/P(VDF-TrFE-CFE)(40/60wt)	~700	~10.7	~90	2	[S2]
PMMA/P(VDF-TrFE)(30/70 wt)	450	10	75	3	[S3]
PVDF/ArPTU (90/10vol)	700	10.8	83	4	[S4]
ArPTU/P(VDF-TrFE-CTFE)(15/85wt)	700	19.2	85	5	[S5]
P(VDF-TrFE-CFE)/PMMA/P(VDF-TrFE-CFE) (5/90/5 vol)	400	9.7	78	6	[S6]
P(VDF-HFP)/PMMA/P(VDF-HFP) (35/30/35 vol)	440	~20.3	~84	7	[S7]
PVDF/DE/PVDF (40/20/40)	438	20.92	72	8	[S8]
P(VDF-HFP)/P(VDF-TrFE-CFE) (50/50 vol) (16 layers)	637.5	20	~85	9	[S9]
P(VDF-HFP)/P(TFE-HFP-VDF) (66.6/33.3 vol) (9 layers)	638	15.5	80.4	10	[S10]
P(VDF-TrFE-CFE)/BNNS 12 wt% composite	650	20.3	~78	11	[S11]
chitin/BNNS 6 wt% composite	451	8.67	~88	12	[S12]
composite film interlayered with BNNSs	612	14.3	~70	13	[S13]
PVDF/BZT/BNNS	678	23.4	83	14	[S14]
P(VDF-TrFE-CFE)/BNNS-BT	527	15.82	~78	15	[S15]
PMF-3L	501.85	7.521	85	16	[S16]
rGO-PI/BNNS-PI composites	74.4	14.2	~65	17	[S17]
Pure PI	443	3.33	85	18	
Pure PEI	599	5.56	94	19	
Pure PP	672	4.97	97	20	
Pure PET	660	6.8	96.3	21	
PET/BNNS	735	8.77	96.5	This work	

Table S2 E_b of PI, PEI, PP, PET and PET/BNNS-2.67 vol% at different temperatures

Sample	Room Temperature	80°C	Reduction	100°C	Reduction	120°C	Reduction
PI	443.42	435.66	1.75%	417.04	5.95%	395.42	10.82%
PEI	559.69	551.32	1.49%	531.72	5.00%	507.61	9.30%
PP	673.84	620.57	7.91%	539.18	19.98%	517.56	23.19%
PET	659.91	630.24	4.50%	623.19	5.56%	595.89	9.70%
PET/BNNS-2.67 vol.%	737.60	682.20	7.51%	675.44	8.43%	664.59	9.90%

Supplementary References

[S1] X. Q. Chen, Y. Wang, D. L. He, Y. Deng, Enhanced dielectric performances of polypropylene films via polarity adjustment by maleic anhydride-grafted polypropylene. J. Appl. Polym. Sci. **134**, 45029 (2017). https://doi.org/10.1002/app.45029

[S2] F. H. Liu, Z. Y. Li, Q. Wang, C. X. Xiong, High breakdown strength and low loss binary polymer blends of poly(vinylidene fluoride-trifluoroethylene-chlorofluoroethylene) and poly(methyl methacrylate). Polym. Adv. Technol. **29**, 1271-1277 (2018). https://doi.org/10.1002/pat.4238

[S3] W. M. Xia, Q. P. Zhang, X. Wang, Z. C. Zhang, Electrical energy discharging performance of poly(vinylidene fluoride-co-trifluoroethylene) by tuning its ferroelectric relaxation with polymethyl methacrylate. J. Appl. Polym. Sci. **131**, 40114 (2013). https://doi.org/10.1002/app.40114

[S4] W. P. Li, L. Jiang, X. Zhang, Y. Shen, C. W. Nan, High-energy-density dielectric films based on polyvinylidene fluoride and aromatic polythiourea for capacitors. J. Mater. Chem. A **2**, 15803-15807 (2014). https://doi.org/10.1039/C4TA03374D

[S5] H. Zhu, Z. Liu, F. H. Wang, Improved dielectric properties and energy storage density of poly(vinylidene fluoride-co-trifluoroethylene-co-chlorotrifluoroethylene) composite films with aromatic polythiourea. J. Mater. Sci. **52**, 5048 (2017). https://doi.org/10.1007/s10853-016-0742-6

[S6] M. J. Feng, T. D. Zhang, C. H. Song, C. H. Zhang, Y. Zhang et al., Improved energy storage performance of all-organic composite dielectric via constructing sandwich structure. Polymers **12**, 1972 (2020). https://doi.org/10.3390/polym12091972

[S7] J. Chen, Y. F. Wang, Q. B. Yuan, X. W. Xu, Y. J. Niu et al., Multilayered ferroelectric polymer films incorporating low-dielectric-constant components for concurrent enhancement of energy density and charge–discharge efficiency. Nano Energy **54**, 288-296 (2018). https://doi.org/10.1016/j.nanoen.2018.10.028

[S8] J. Chen, Y. F. Wang, X. W. Xu, Q. B. Yuan, Y. J. Niu et al., Sandwich structured

poly(vinylidene fluoride)/polyacrylate elastomers with significantly enhanced electric displacement and energy density. J. Mater. Chem. A **6**, 24367-24377 (2018). https://doi.org/10.1039/C8TA09111K

[S9] J. Y. Jiang, Z. H. Shen, J. F. Qian, Z. K. Dan, M. F. Guo et al., Ultrahigh discharge efficiency in multilayered polymer nanocomposites of high energy density. Energy Storage Mater. **18**, 213-221 (2019). https://doi.org/10.1016/j.ensm.2018.09.013

[S10] Y. S. Li, S. Cheng, S. J. Wang, C. Yuan, Z. Luo et al., Multilayered ferroelectric polymer composites with high energy density at elevated temperature. Compos. Sci. Technol. **202**, 108594 (2021). https://doi.org/10.1016/j.compscitech.2020.108594

[S11] Q. Li, G. Z. Zhang, F. H. Liu, K. Han, M. R. Gadinski et al., Solution-processed ferroelectric terpolymer nanocomposites with high breakdown strength and energy density utilizing boron nitride nanosheets. Energy Environ. Sci. **8**, 922-931 (2015). https://doi.org/10.1039/C4EE02962C

[S12] J. Y. Wang, H. Chen, X. Q. Li, C. G. Zhang, W. C. Yu et al., Flexible dielectric film with high energy density based on chitin/boron nitride nanosheets. Chem. Eng. J. **383**, 123147 (2020). https://doi.org/10.1016/j.cej.2019.123147

[S13] Y. K. Zhu, Y. J. Zhu, X. Y. Huang, J. Chen, Q. Li et al., High energy density polymer dielectrics interlayered by assembled boron nitride nanosheets. Adv. Energy Mater. 9, 1901826 (2019). https://doi.org/10.1002/aenm.201901826

[S14] J. Y. Jiang, Z. H. Shen, X. K. Cai, J. F. Qian, Z. K. Dan et al., Polymer nanocomposites with interpenetrating gradient structure exhibiting ultrahigh discharge efficiency and energy density. Adv. Energy Mater. **9**, 1803411 (2019). https://doi.org/10.1002/aenm.201803411

[S15] Y. S. Li, Y. Zhou, Y. J. Zhu, S. Cheng, C. Yuan et al., Polymer nanocomposites with high energy density and improved charge–discharge efficiency utilizing hierarchically-structured nanofillers. J. Mater. Chem. A **8**, 6576-6585 (2020). https://doi.org/10.1039/D0TA01380C

[S16] S. B. Sun, Z. C. Shi, L. Sun, L. Liang, D. Dastan et al., Achieving concurrent high energy density and efficiency in all-polymer layered paraelectric/ferroelectric composites via introducing a moderate layer. ACS Appl. Mater. Interfaces **13**, 27522-27532 (2021). https://doi.org/10.1021/acsami.1c08063

[S17] F. M. Guo, X. Shen, J. M. Zhou, D. Liu, Q. B. Zheng et al., Highly thermally conductive dielectric nanocomposites with synergistic alignments of graphene and boron nitride nanosheets. Adv. Funct. Mater. **30**, 1910826 (2020). https://doi.org/10.1002/adfm.201910826
